# Urban Stormwater Runoff: A New Class of Environmental Flow Problem

**DOI:** 10.1371/journal.pone.0045814

**Published:** 2012-09-19

**Authors:** Christopher J. Walsh, Tim D. Fletcher, Matthew J. Burns

**Affiliations:** 1 Department of Resource Management and Geography, The University of Melbourne, Parkville, Victoria, Australia; 2 Department of Civil Engineering and Monash Water for Liveability, Monash University, Victoria, Australia; Argonne National Laboratory, United States of America

## Abstract

Environmental flow assessment frameworks have begun to consider changes to flow regimes resulting from land-use change. Urban stormwater runoff, which degrades streams through altered volume, pattern and quality of flow, presents a problem that challenges dominant approaches to stormwater and water resource management, and to environmental flow assessment. We used evidence of ecological response to different stormwater drainage systems to develop methods for input to environmental flow assessment. We identified the nature of hydrologic change resulting from conventional urban stormwater runoff, and the mechanisms by which such hydrologic change is prevented in streams where ecological condition has been protected. We also quantified the increase in total volume resulting from urban stormwater runoff, by comparing annual streamflow volumes from undeveloped catchments with the volumes that would run off impervious surfaces under the same rainfall regimes. In catchments with as little as 5–10% total imperviousness, conventional stormwater drainage, associated with poor in-stream ecological condition, reduces contributions to baseflows and increases the frequency and magnitude of storm flows, but in similarly impervious catchments in which streams retain good ecological condition, informal drainage to forested hillslopes, without a direct piped discharge to the stream, results in little such hydrologic change. In urbanized catchments, dispersed urban stormwater retention measures can potentially protect urban stream ecosystems by mimicking the hydrologic effects of informal drainage, if sufficient water is harvested and kept out of the stream, and if discharged water is treated to a suitable quality. Urban stormwater is a new class of environmental flow problem: one that requires reduction of a large excess volume of water to maintain riverine ecological integrity. It is the best type of problem, because solving it provides an opportunity to solve other problems such as the provision of water for human use.

## Introduction

Humanity faces a major challenge to provide the world's growing population with reliable and affordable water supplies, while protecting the ecological integrity of freshwater ecosystems [1]. The concept of environmental flows has developed to meet this challenge, with the aim of identifying the critical elements of flow regimes that should be retained or restored [2]. Environmental flow assessment and provision has to date focussed mainly on mitigation of the effects of water extraction, but increasingly, changes to the flow regime resulting from land-use changes are being considered [3], [4].

Of all land-uses, urbanization arguably causes the largest changes to volumes and patterns of flow running from catchments to streams and rivers. The urban water system has three elements; water supply, wastewater and stormwater. Managers of urban water resources focus primarily on water supply imported from distant sources (usually rural rivers), or from groundwater, distributed through a network of water supply pipes, and on wastewater exported through a second network to treatment plants. While the imported water supply and exported wastewater of urban areas substantially alters the water balance of cities [5], in most modern cities with separate stormwater drainage and sanitary sewerage systems, these elements of the urban water system bypass, and therefore have relatively little influence on, the flow regime of streams and rivers of the city themselves. It is the third element of the urban water system–stormwater (runoff from impervious surfaces during and immediately after wet weather)–, generally of a similar volume to imported water [6], but rarely considered by water resource managers, that has the largest effect on flow regimes of urban streams and rivers.

**Figure 1:**
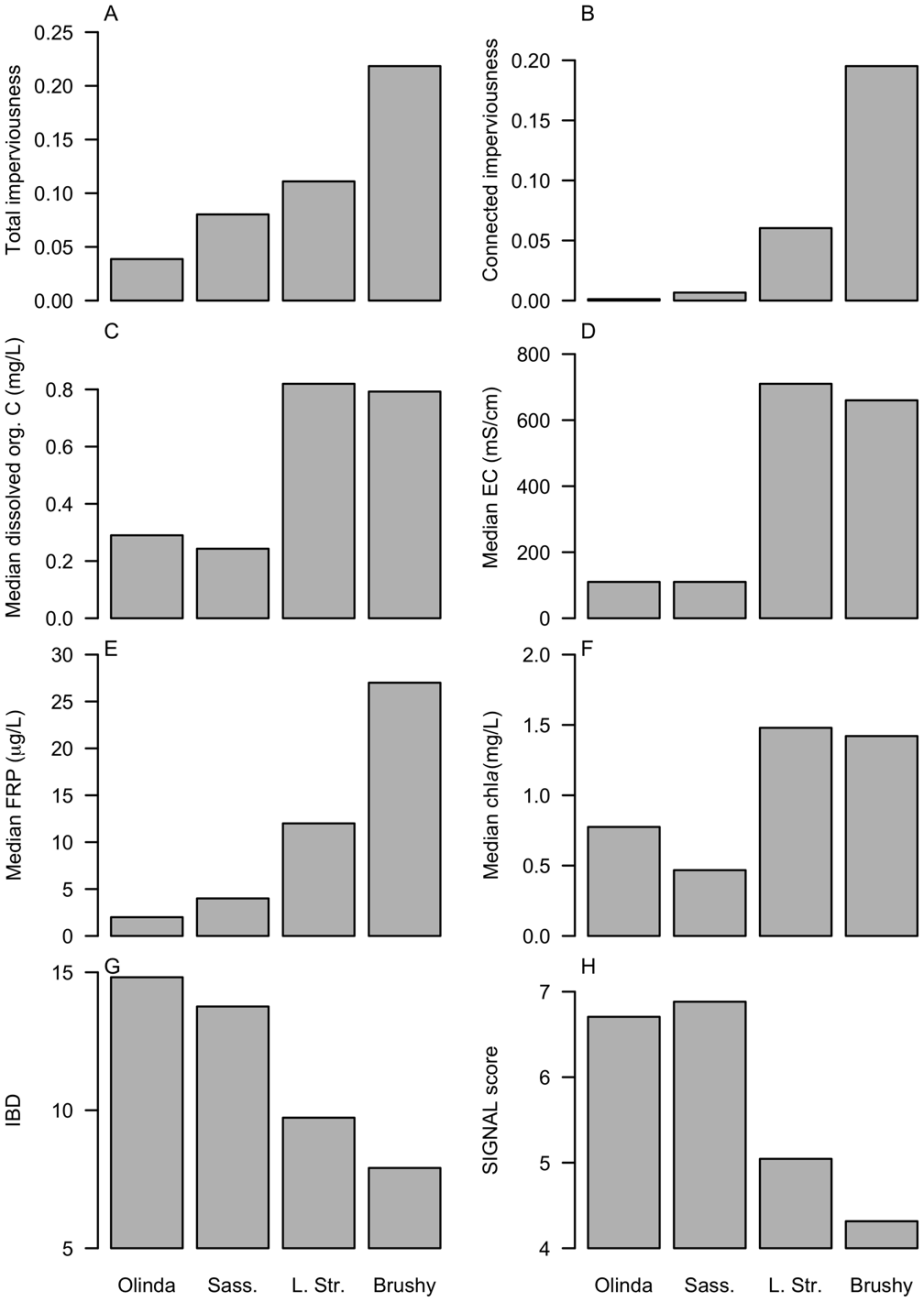
Catchment urbanization measures and in-stream ecological indicators for four contrasting streams in eastern Melbourne. (A) Total and (B) connected imperviousness (estimated for 2004). Median 2001–2002 (C) dissolved organic carbon, (D) electrical conductivity, (E) filterable reactive phosphorus. (F) Mean 2001–2002 median benthic chlorophyll *a*, as an estimate of algal biomass. (G) Indice biologique diatomée (IBD) [53]. (H) SIGNAL score [54] for stream-edge samples. IBD and SIGNAL are indices based on diatom species and macroinvertebrate families, respectively, weighting each taxon by their sensitivity to pollution. Adapted from Walsh et al. [18], with minor revision to Little Stringybark Creek values from 2004 aerial photos and ground-truthing). Sass.  =  Sassafras Creek, L. Str.  =  Little Stringybark Creek.

It has long been recognized that covering land with impervious surfaces, such as roofs and roads, reduces both the volume of water infiltrating into soils and the volume of water lost to the air through evapotranspiration, thus increasing the volume of runoff following rain events [7]. These changes to the water balance are exacerbated, and their effects transferred to streams and rivers, by stormwater drainage systems: a third network of pipes under cities (that have separate sewers), which is designed to minimize flood risk, by efficiently draining all runoff from impervious surfaces directly to the nearest receiving water [7]. Here we term such drainage systems, that directly connect impervious surfaces to a receiving water (via gutters, pipes and perhaps sealed channels), conventional stormwater drainage [8].

**Figure 2:**
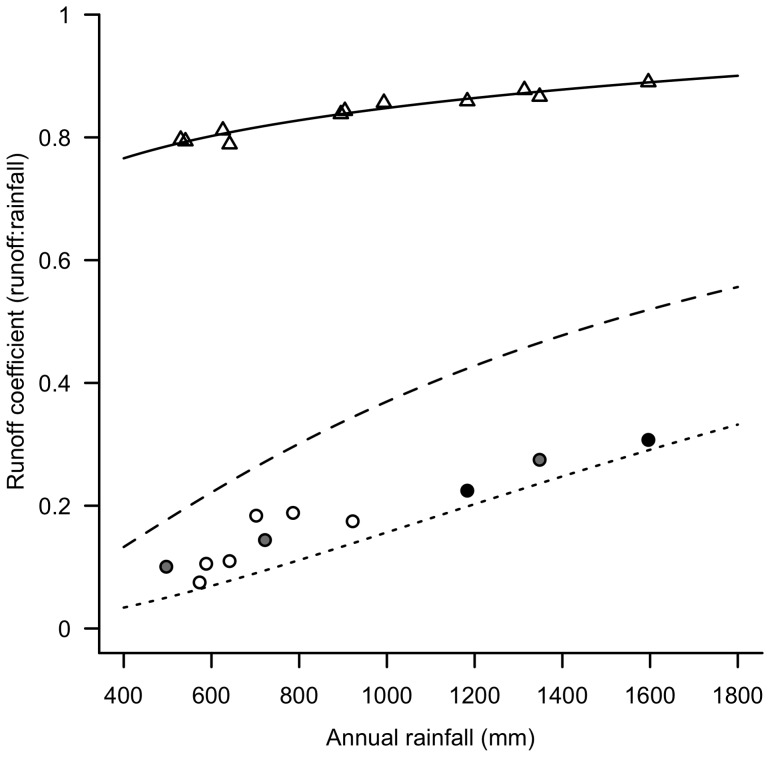
Impervious runoff and streamflow coefficients for the Melbourne region. Estimated annual runoff coefficients (C) from impervious surfaces (open triangles) from sites across the Melbourne region as a function of mean annual rainfall (MAR). Regression line: C = 0.230+0.206×log_10_(MAR). *R* ^2^ = 0.94. Annual streamflow coefficients from 11 streams with forested (closed circles), grassland (open circles) or mixed forested and grassland catchments (grey circles) across the Melbourne region as a function of mean annual rainfall. The lines surrounding these stream points are the relationships between streamflow derived by Zhang et al. [20] for grassland (dashed curve) and forested catchments (dotted curve) of the world.

Conventional stormwater drainage has been identified as a primary driver of the commonly observed, severe degradation of stream ecosystems in urban catchments [9], [10]. Such systems send polluted stormwater to receiving waters every time there is sufficient rain to generate runoff from impervious surfaces, greatly increasing the frequency of hydraulic and water quality disturbances to streams [11]. The large changes to the volume and pattern of flow caused by urban stormwater drainage systems point to urban stream degradation being, in large part, a hydrologic problem. Its solution should therefore be informed by approaches to environmental flow management.

**Table 1:** Flow and rainfall gauging stations used in the water balance analyses, with period of data used.

Gauge	Stream	Years	Vegetation^3^	Rainfall^4^
406213^1^	Campaspe32	1985–1994	Pasture, crops	
407214^1^	Creswick Creek	1985–1994	Mixed	
407253^1^	Piccaninny Creek	Long term	Mixed	
406224^1^	Mt. Pleasant Creek	1985–1994	Pasture	
406235^1^	Wild Duck Creek	1985–1994	Pasture, crops	
406262^1^	Axe Creek 33	1990–1994	Pasture, crops	
406200^1^	Coliban River	1985–1993	Pasture, crops	
228207A^2^	Bunyip River at Headworks	1994–1996	Forest	x
229627^2^	Merri Ck	1994–1996	Pasture	x
229215B^2^	Woori Yallock Ck	1994–1996	Mixed	x
229690^2^	Olinda Ck at Mt Evelyn	1994–1996	Forest	x
586097	Mt St Leonard	1954–1999		x
086027	Croydon	1965–1970, 1980–1997		x
086251	Upwey	1968–1976, 1978–2001		x
086071	Melbourne CBD	1970–2001		x
086282	Melbourne airport	1970–2001		x
587030	Werribee	1968–1990		x

1 Streams outside the Melbourne region, for which streamflow coefficients were derived by Jolly et al. [22] and catchment vegetation classes were assigned by Zhang et al. [19].

2 Streams in the Melbourne region, for which streamflow coefficients were derived using Melbourne Water flow gauge data and Bureau of Meteorology rainfall data for 1994–1996. Catchment vegetation classes were estimated from 2001 aerial imagery.

3 Catchment vegetation class is indicated for flow-gauging stations used to calculate streamflow coefficients. ^4^Stations from which rainfall data were used to estimate impervious runoff are indicated in the final column.

Recent considerations of environmental flow requirements for freshwater and estuarine systems have broadened the focus of environmental flow management to include all aspects of land and water use, and seek to integrate water quality with flow management [2]. To date, urban stormwater runoff has primarily been considered a water quality problem [8]. The combined hydrologic and water quality perturbations caused by urban stormwater runoff make the management of urban stormwater an ideal case to develop and apply the new broad conception of environmental flow management [2].

**Figure 3:**
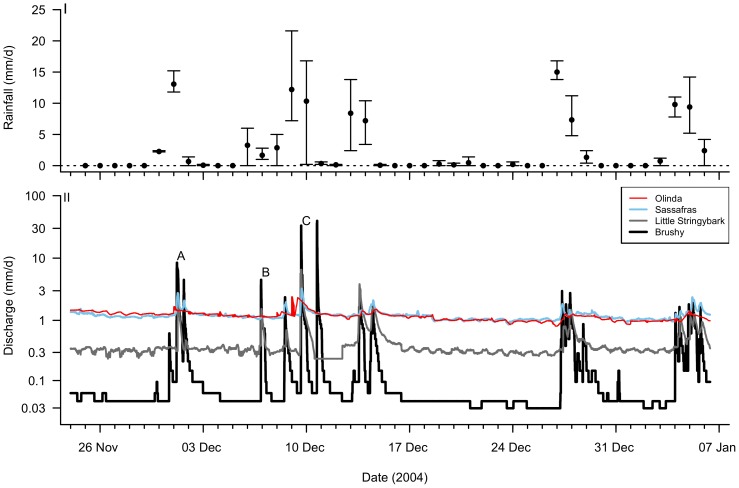
Rainfall and discharge of the four study streams over 43 days in 2004. I. Mean ± range daily rainfall recorded in the three rain gauges that fall within the area bounded by catchments of the streams. II. 5- or 6-minute hydrographs for each stream. Three flow events (A, B and C) are discussed in the text.

Poff et al. [3] developed a framework for determining and implementing this new conception of environmental flow requirements. The framework requires initial hydrologic analyses to identify the nature and degree of flow alteration for each river type (based on hydrologic and geomorphic classifications) followed by an assessment of responses of river biota and ecological processes to flow alteration, a social process for determining ecological objectives, and adaptive implementation and monitoring [3].

**Figure 4:**
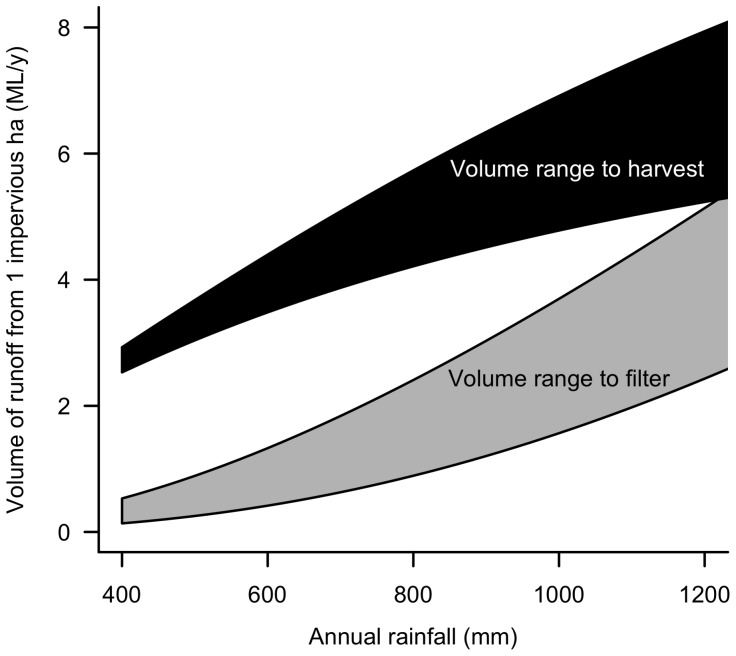
Impervious runoff volume partitioned into lost subsurface flows and lost evapotranspiration. Annual volume of runoff from 1 ha of impervious surface (from the relationship between impervious runoff coefficient and annual rainfall shown in Figure 2), partitioned into two parts: the volume that needs to be passed through infiltration systems (or catchment soils) to restore lost subsurface flows (grey polygon), and the volume that needs to be retained in the catchment and not delivered to the stream (through evapotranspirational loss or through use and export from the catchment through the wastewater stream). For each part, a range is indicated between situations in which the target streamflow is predicted by the grassland curve (more stream flow, less retention in catchment) or by the forest curve (less streamflow, more retention in the catchment) of Zhang et al. [20] (Figure 2).

A primary step in assessing and implementing environmental flows is to estimate how ecologically relevant components of the flow regime are altered by a human activity [3]. Typically, ecologically relevant indicators of hydrologic alteration have been selected by identifying those hydrologic indicators that are well correlated with changes in ecological indicators, and a few studies have applied such an approach in an urban setting. The general conclusion from such studies (e.g. [4], [12]) is that the primary urban-induced hydrologic changes driving ecological degradation centre on the increased frequency and magnitude, and reduced duration of storm flow events, and reduced low flows. While these studies did not explicitly consider water quality, both high and low flows in urban areas are associated with increased pollutant concentrations [13].

**Figure 5:**
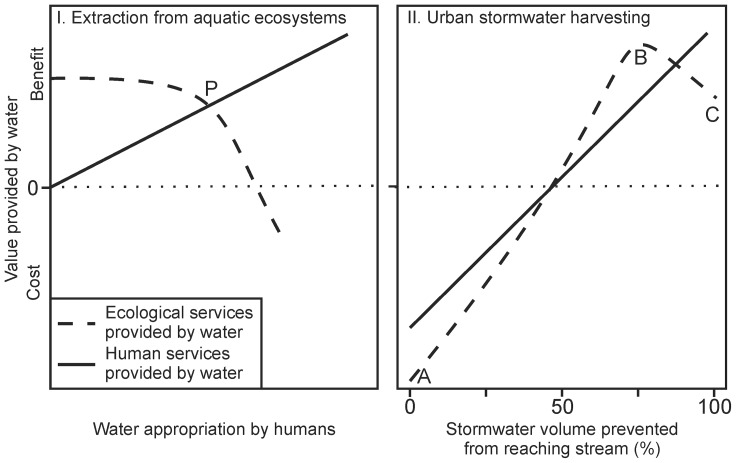
Conceptual graphs of ecological and human value of water. I. The model proposed by Gleick and Palaniappan [47] assumes that any extraction from aquatic ecosystems has a negative ecological impact, predicting a monotonic decline of increasing gradient with greater extraction. The benefits accrued by the human population rise linearly with the volume extracted. Beyond peak ecological water (P) [47], any increase in human benefit is outweighed by reduced ecological benefit. II. illustrates different trends in ecological and human cost and benefit with increasing retention and use of stormwater before it reaches aquatic ecosystems. No stormwater use (A) results in ecological degradation of receiving waters. It also presents greater costs in urban microclimate control and flood mitigation than if stormwater was harvested. Using a volume of stormwater equivalent to the volume lost to evapotranspiration in the pre-urban state (B), if coupled with infiltration systems to restore lost sub-surface flows, provides maximum environmental benefit. Using all available stormwater runoff (C) has an environmental cost by reducing subsurface flow delivery to stream.

When provision of environmental flow requirements is addressed solely through management of water storages and diversions, the mechanisms for hydrologic alteration are usually obvious. However, when the primary environmental flow problem is a result of altered land use, such as urbanization, then an additional step in an environmental flow assessment framework is required: determination of the mechanisms for the hydrologic alteration, and strategies for their mitigation. The ultimate mechanisms by which urbanization degrades streams are manifold, but can usually be attributed to a small number of landscape-scale land-use practices for which alternative management approaches are available [9]. Hydrologists have long recognised that conventional stormwater drainage is the dominant driver of urban-induced hydrologic changes [7], [8], [14], leading ecologists to posit the importance of such systems in driving the ecological degradation of urban stream ecosystems [9].

Only a few studies have attempted to distinguish the effects of urban stormwater drainage on stream ecosystems from the general effects of urban density. Most studies of ecological and hydrologic changes resulting from urbanization have used total impervious coverage or other correlated urban land-use measures as explanatory variables (e.g. [15], [16], [17]), and have therefore been unable to discern if variation in ecological response could have been caused by differences in stormwater drainage systems. Differences that could alter the response of stream ecosystems and their flow regimes include the proportion of impervious areas that are connected to conventional drainage systems, and the hydraulic efficiency of alternative drainage systems (i.e. the degree to which water and attached pollutants are retained or attenuated as they are transferred downstream). Studies of streams across urban areas with differing extent of conventional urban stormwater drainage have shown that streams with substantial catchment urbanization can retain ecological structure and function more typical of streams with undeveloped catchments, if their urban areas lack conventional stormwater drainage systems [18]. In contrast, streams with similar levels of catchment urbanization (as measured by total imperviousness), but in which most urban surfaces are drained by conventional stormwater drainage, exhibit poor ecological condition.

In this paper, we use evidence of ecological response to different stormwater drainage systems to develop methods for input to environmental flow assessment. We do not aim to conduct a full environmental flow assessment, but to demonstrate a process for identifying the nature of hydrologic change resulting from conventional urban stormwater runoff, and the mechanisms by which such hydrologic change is prevented in streams where ecological condition has been protected. We propose these as important steps in environmental flow assessment for stormwater management.

Unlike the usual environmental flow problem of needing to allocate a reduced volume of water to the environment, urban stormwater runoff presents a problem of increased runoff volume, which should be prevented from reaching receiving streams and thus could be used by humans. We quantify the magnitude of this problem by comparing streamflow volumes from a range of undeveloped catchments in the Melbourne region with the volumes that would run off impervious surfaces in those catchments, and use a published global analysis to demonstrate the generality of these findings. Our analyses reveal urban stormwater as a new class of environmental flow problem: one that requires reduction of a large excess volume of water to maintain riverine ecological integrity. Urban stormwater runoff is thus revealed as the best type of problem, because solving it provides an opportunity to solve other problems such as the provision of water for human use in cities.

## Materials and Methods

Our analysis comprises two parts. We first compare hydrographs of four streams of contrasting catchment urban density and stormwater drainage infrastructure. We use these data to contrast the severe alterations to flow patterns resulting from conventional stormwater drainage, to the conservation of more natural flow patterns afforded by informal drainage. We thus link these hydrologic patterns with previously reported strong correlation between stormwater drainage connection and ecological indicators to infer that the observed changes to flow regime are likely important drivers of observed ecological degradation. Because such a short-term hydrographic analysis does not allow a valid assessment of the urban water balance, we use a second approach to assess the influence of conventional urban stormwater drainage on streamflow coefficients. We compare streamflow coefficients for streams of the world [19], [20] as a function of catchment rainfall and vegetation, validated with coefficients calculated for streams of the Melbourne region with a long flow record. We compare these to impervious runoff coefficients estimated from long-term rainfall records.

### Determining the effect of conventional stormwater drainage on flow regimes

We first consider four of the small streams in eastern Melbourne, Victoria, Australia, studied by Walsh et al. [18], each with some level of catchment urbanization, but two of which almost completely lack conventional stormwater drainage and two for which most urban areas are drained conventionally (Figure 1). The two streams with near-zero connected imperviousness, Olinda and Sassafras creeks, were in good ecological condition: they shared comparably low pollutant concentrations and algal biomass, and supported algal and invertebrate assemblages comparable to undisturbed forested streams of the region (Figure 1). Little Stringybark Creek, which has only marginally higher total imperviousness than Sassafras Creek, but substantially higher connected imperviousness, was in poor ecological condition, like the even more urban Brushy Creek (Figure 1).

Most of the impervious surfaces of Little Stringybark and Brushy creeks drain to conventional drainage systems, as evidenced by the small differences between total and connected imperviousness (Figure 1A, B). In contrast, the very low levels of connected imperviousness in Sassafras and Olinda result from the buildings and roads of these catchments being almost all drained informally: roofs generally drain to surrounding gardens or to rainwater tanks, and almost all sealed roads lack curbs, and drain either into the surrounding forest or to earthen or vegetated swales. The main road of Sassafras township, at the head of the Sassafras catchment, is conventionally drained by curb and channel, leading to a pipe which drains to the forested hillslope below, ∼150 m upslope from the creek. In 2004, the outlet of the pipe distributed flow across the forest floor, permitting most flows from the pipe to infiltrate into forest soils.

The catchments of all four streams are dominated by urban land or forest, with little agriculture, except for a small area of horticulture in the Olinda Creek catchment, and low-intensity grazing in rural-residential areas of Little Stringybark Creek. Our conclusions are not influenced by abstraction patterns: there is no abstraction from Brushy or Little Stringybark creeks, and only very minor abstraction in Sassafras and Olinda Creek (mean licenced volume of 0.01 and 0.03 mm/d respectively: source Melbourne Water). See [21] for further details on the catchment characteristics of the four streams.

To assess the flow regime in the four sites we used 6-min time-step flow data from a permanent flow gauge installed ∼100 m upstream of the sampling site on Brushy Creek (Melbourne Water station 229249: www.melbournewater.com.au), and a limited record of water depth collected at the other three sites (using Odyssey™ capacitance water level probes–Dataflow Systems, Christchurch, New Zealand–in 44-mm diameter polyvinyl-chloride stilling wells, logging at 5-min intervals). Concurrent data without gaps for all four streams were only available for the period 24 Nov 2004–6 Jan 2005 (43 days).

Discharge of Sassafras and Little Stringybark creeks was estimated manually using either a CMC 20 current meter counter or a Marsh-McBirney flow-mate™velocity meter, 6–10 times at each site over Nov 2004–Mar 2005, and relationships between discharge and simultaneous depth logger readings were derived. Discharge in Olinda Creek was estimated using a quadratic relationship between discharge recordings from a permanent flow station downstream (Melbourne Water Station 229260, catchment area 23 km^2^ compared to 9 km^2^ for our site, with similar catchment land use) and corresponding depth logger readings. All discharge estimates were normalized by catchment area, to be expressed as mm/d.

Discharge patterns in the four streams were compared to the daily rainfall recorded at the two Melbourne Water flow gauges and at the Bureau of Meteorology (www.bom.gov.au) pluviograph on Mt Dandenong (Station 086243). The three rainfall stations were within the area bounded by the four study catchments.

While the period of record considered here is short, it encompassed several rain events of a wide range of sizes (in which rain was relatively evenly spread across the catchments), and two inter-event periods that permit an assessment of differences and similarities in hydrologic response of the four streams.

### Quantifying the increased volume of urban stormwater runoff

To assess the increase in runoff volume generated by impervious surfaces in the Melbourne region, we compared streamflow coefficients (mean annual discharge depth divided by mean annual rainfall) of undeveloped, unregulated streams in the region with impervious runoff coefficients (mean annual runoff depth from an impervious area divided by mean annual rainfall). The difference between the streamflow coefficient and impervious runoff coefficient of a site represents the loss of evapotranspiration and equates to the excess runoff generated by a given area of impervious surface above the volume that would have contributed to streamflow in the pre-urban state (the lost infiltration flows). The analyses were undertaken using streamflow and rainfall data from across the Melbourne region (Table 1), spanning the range of mean annual rainfall in the region (400–1800 mm/y, although the wettest urban areas receive 1200 mm/y).

The range of streamflow coefficients for streams and rivers of the Melbourne region in the absence of urbanization was estimated using the relationships derived by Zhang et al. [19], [20] that predict the proportion of mean annual rainfall lost to evapotranspiration (and conversely, the proportion that became stream flow, assuming no changes in soil water storage over the long-term and no loss to deep seepage [20]) as a function of catchment vegetation cover (grassland or forest) and rainfall (Figure 2). To confirm that measured streamflow coefficients for Melbourne streams fall within the predicted bounds for grassland and forested catchments, we plotted streamflow coefficients calculated by Jolly et al. [22], for seven undeveloped, unregulated streams within 200 km of Melbourne (Table 1). We also determined streamflow coefficients for four Melbourne Water flow gauges on streams with undeveloped, unregulated catchments within the Melbourne region. For each of these gauges, weighted mean catchment rainfall was estimated using dynamically assigned Thiessen polygons around all available daily rain gauge data (Bureau of Meteorology) for 1994–1996 (Table 1).

Runoff from impervious surfaces was estimated from daily rainfall data from 11 gauges across the Melbourne region (Table 1), assuming an initial loss of 1 mm/d (e.g. [23]). Impervious runoff coefficients were calculated by dividing the sum of estimated impervious runoff by the sum of rainfall over the period. To compare impervious runoff coefficients and streamflow coefficients in undeveloped catchments, the relationship between impervious runoff coefficient and mean annual rainfall (log-transformed to reduce leverage of high values) was determined by linear regression. The increase in impervious runoff was estimated using the range of differences between this line of best fit for impervious runoff coefficients and the two curves derived by Zhang et al. [20] for streamflow coefficients in grassland and forested catchments (Figure 2).

## Results

### Determining the effect of conventional stormwater drainage on flow regime

Three major differences in the four hydrographs (Figure 3) are likely to signify ecologically important changes to the flow regime:

1. Olinda and Sassafras creeks, which had similarly low connected imperviousness (Figure 1), had similarly high baseflow, while Little Stringybark Creek, which had substantially higher connected imperviousness, but similar total imperviousness to Sassafras Creek (Figure 1), had lower baseflow. Brushy Creek, which had higher connected imperviousness again (Figure 1), had even lower baseflow;.
2. Discharge increased only slightly in response to rain events of 10–20 mm in Olinda and Sassafras creeks (e.g. events A and C, Figure 3), with a slightly larger response in the latter stream, usually not exceeding an increase in discharge of 2–3 times. In contrast, such events caused an increase in discharge of an order of magnitude in Little Stringybark Creek, and of 2–3 orders of magnitude in Brushy Creek.
3. Discharge did not change in Olinda and Sassafras creeks following rain events of <5 mm/d (e.g. event B, Figure 3), compared to large increases in discharge in Little Stringybark and Brushy creeks.

### Quantifying the increased volume of urban stormwater runoff

Streamflow coefficients in and around the Melbourne region consistently fell within the bounds of the relationships derived by Zhang et al. [20] for forested (lower bound) and grassland (upper bound) catchments (Figure 2). While the predicted effect of vegetation on streamflow coefficient is not evident from the small number of points for the Melbourne region, the two curves provide useful bounds for estimating streamflow from undeveloped, unregulated catchments.

Impervious runoff coefficients were well predicted by mean annual rainfall (*R* ^2^ = 0.94), and were consistently much higher than streamflow coefficients in undeveloped streams (Figure 2). More importantly, the difference between streamflow and impervious runoff coefficients equates to an excess volume of runoff from 1 ha of impervious surface of 2.6–3.0 ML/y in catchments with mean annual rainfall of 400 mm rising to 5.1–7.8 ML/y in catchments with 1200 mm/y (Figure 4).

## Discussion

### Mechanisms by which informal drainage protects streams

The similarity of flow regimes in Olinda and Sassafras creeks (Figure 3) and their good water quality and ecological condition (Figure 1) demonstrates the potential for streams with substantial catchment urbanization to retain important elements of the flow regime and water quality that are likely to be required for the protection of in-stream ecological values.

The lack of conventional stormwater drainage in these two catchments points to several mechanisms that can be replicated by appropriate stormwater management technologies to retain or restore components of the flow regime. The clear driver of increased frequency and magnitude of high flows observed in Little Stringybark and Brushy creeks, but not the other two streams, is impervious runoff directed to the stream through pipes.

The informal drainage that dominates the Sassafras and Olinda catchments permits the retention (including some harvesting) and infiltration (and subsequent uptake and loss by the large area of downslope plants) of water from frequent, small-to-moderate storm flows [18], explaining the lack of streamflow response to events of <5 mm/d in Sassafras and Olinda creeks. In contrast, the conventional stormwater drainage systems of Little Stringybark and Brushy creeks pass runoff and its associated pollutants directly to the streams in all events large enough to elicit runoff from impervious surfaces (typically >1 mm/d, e.g. [23], although runoff from pitched roofs is typically initiated after as little as 0.5 mm [24]), explaining both the increased frequency and magnitude of high flows observed in these two streams.

Reduced infiltration resulting from coverage of impervious surfaces and lined drainage systems in Little Stringybark and Brushy creeks is most likely exacerbated by soil changes that occur through urbanization. The residential gardens of Little Stringybark and Brushy catchments are typically less treed than those of Sassafras and Olinda, and compaction of topsoils is likely to be more widespread. The resultant loss of soil storage capacity is likely to be another important driver of reduced baseflows in these catchments [25].

However, pervious areas with reduced soil storage capacity are unlikely to be large contributors to increased frequency or magnitude of high flows. Like informally drained impervious surfaces, runoff from such surfaces is unlikely to find its way into stormwater drains in most rain events. Instead, water is likely to flow to a downslope area with greater infiltration capacity, augmenting subsurface flow. Studies of hillslope storm hydrology of forested catchments have generally found subsurface flow to be the dominant pathway (e.g. [26]). Hillslopes form complex mosaics with differing soil porosity, meaning that any overland flow generated from intense rain events on a hillslope is likely to be re-absorbed into the soils downslope before reaching a stream [27].

In contrast, all urban stormwater delivered through conventional drainage systems is delivered to the receiving water unfiltered, through pipes, resulting in more frequent, larger flood peaks of shorter duration and of poor quality. The challenge for urban stormwater managers is therefore to mimic the natural hillslope hydrology of forested catchments, so that impervious runoff is delivered to streams with the appropriate temporal pattern, volume and quality.

### Environmental flow standards for stormwater management

This assessment of the hydrologic differences between streams with informal catchment drainage (that retain good ecological condition) and streams with conventional stormwater drainage (that are degraded ecologically), allows the identification of environmental flow standards for the protection of streams from urban stormwater runoff. The first requirement of such standards is the provision of flow through filtration systems (or catchment soils) that mimics the temporal pattern, quantity and quality of subsurface flows in undeveloped catchments.

Secondly, the systems should be sized so that they overflow and spill to conventional drainage systems or streams only infrequently: ideally at a frequency similar to that of storm events large enough to initiate widespread overland flow on hill slopes of the undeveloped catchment.

In sparsely-to-moderately urbanized catchments such as Olinda and Sassafras creeks, informal drainage provides protection to the streams because the catchments retain sufficient areas of intact forest soils downslope that can absorb the additional runoff from urban surfaces. In more densely urbanized areas, the magnitude of flow increases and a lack of downslope soil storage (and coupled evapotranspiration through vegetation) will likely require constructed retention and treatment technologies to adequately replicate forested hillslope processes. Such techniques are likely to involve some form of infiltration.

Infiltration systems can be designed to retain and treat storm flows to substantially reduce the frequency of unfiltered runoff from impervious surfaces, while helping to restore baseflows [28], [29]. However, because impervious surfaces increase the volume of stormwater runoff and reduce vegetation cover (reducing capacity to lose water to evapotranspiration), very large infiltration systems will be needed to adequately retain storm flows, unless a large proportion of stormwater runoff is harvested. Without harvesting runoff for uses that either result in the water being lost to the wastewater stream through the (separate) sanitary sewer, or to the air through evapotranspiration by urban vegetation, the amount of infiltration required to match pre-development runoff frequency will result in infiltrated (filtered) flows greater than the volume that fed streamflow in the pre-urban state, resulting in increased streamflow volumes. Ideally, across urban Melbourne where mean annual rainfall varies from 400 to 1200 mm/y, only a minority of stormwater runoff should be delivered to the stream (as filtered flows), with the rest being harvested or lost to evapotranspiration (Figure 4).

This therefore points to a third requirement of an environmental flow standard for stormwater management: harvesting of an appropriate volume of stormwater runoff to prevent it from reaching the receiving water. This marks urban stormwater as a unique water resource: one for which human use provides environmental benefit.

### Stormwater management technologies to meet environmental flow standards

The volume of excess urban stormwater runoff in Melbourne corresponds to a large proportion of the city's total water demand, which is currently met almost completely by water from rivers in forested catchments outside the city. The total water demand for Melbourne in 2004–2005 was equivalent to 0.14 ML/y per person [30], while the mean impervious area per person in 2004 was 266 m^2^ (total impervious area 964 km^2^, Melbourne Water, unpubl. data, population 3.63 million [31]). Excess runoff from an impervious area of this size in the driest part of Melbourne equals 57% of the total demand (the upper bound of the black polygon for annual rainfall of 400 mm in Figure 4 = 3 ML/y excess runoff from 1 ha, or 0.08 ML/y from 266 m^2^), while in the wettest part of Melbourne excess runoff from an equivalent impervious area produces 147% of total demand (upper bound of black polygon for annual rainfall of 1200 mm in Figure 4). Such large volumes of excess runoff relative to demand, in the presence of existing water supplies, make adequate retention to protect stream ecosystems challenging.

In many cities of the world, with higher population densities than Melbourne and inadequate water supply systems, water demand is likely to be sufficient to allow harvesting of all excess urban stormwater runoff. In low-density cities such as Melbourne, use of all excess runoff is more challenging, but new and existing technologies for stormwater retention make such retention feasible, while the potential social and environmental benefits of using stormwater to improve the urban landscape and amenity provide incentives to create new uses for stormwater.

Household-scale harvesting (typically of roofwater) is already widely practiced in many places around the world. Collection, storage, distribution and treatment of urban stormwater at larger scales will be necessary to allow adequate harvesting of excess runoff from areas with low demands and distribution to areas with high demands. Such technologies are well developed, requiring storage volumes that are feasible in most urban settings [32]. Substantial challenges remain, however, in the development of cost-effective techniques for the treatment of stormwater [33], [34].

Technologies for stormwater infiltration, such as infiltration basins and trenches, soakaway pits, bioretention systems (rain gardens) and porous pavements are well developed [28], [35]. Rain gardens use vegetated soil media to improve stormwater quality [36], attenuate flow rates [37] and, depending on the design, promote infiltration and evapotranspiration [38]. Where infiltration is not possible, they can be built with an underdrain which discharges treated water directly to the stormwater system (and thus the receiving water), at a rate controlled to match the pre-development low-flow regime [37]. However, to mimic the type of highly attenuated flow regimes observed for Sassafras and Olinda Creek (Figure 3), large detention storage is required.

Typically, infiltration-based systems need to have an area of 2–5% of the upstream impervious area, depending on climate and soil type, to retain enough stormwater to permit replication of infiltration flows that would have occurred in the pre-urban context [38]. Infiltration systems can provide other benefits by forming part of green open space, while porous pavements can form other useable urban surfaces, but with less evapotranspiration loss.

Importantly, infiltration techniques alone are unlikely to achieve the stormwater retention necessary to return streamflow volumes to near natural levels (evapotranspiration losses in infiltration systems will not be enough to mimic pre-development catchment-wide evapotranspiration losses). Integration of harvesting and infiltration-based techniques to restore both high- and low-flow hydrology towards their natural levels is necessary, and can be achieved by simple methods such as using a proportion of harvested storage volumes for passive landscape irrigation. Such systems can reduce overflow frequency and increase evapotranspiration losses and infiltration, with little effect on harvested yields [39].

Existing stormwater management technologies are therefore capable of providing environmental flows to protect stream ecosystems in urban areas. The primary impediments to implementation of such technologies for the protection of streams are social and political [40]. A particular barrier is that the unique nature of the stormwater problem (as an environmental flow) and opportunity (as a water resource) is largely unrecognised in the environmental flow literature, and among urban water managers.

### Urban stormwater as an environmental flow problem and an unrealized water resource

Most environmental flow problems arise from water being extracted for human use: the challenge for environmental flow researchers and practitioners in such situations is how to distribute the remainder for maximum environmental benefit [2]. This focus on extraction of water from aquatic ecosystems, compounded with the tendency for water resource managers to prefer centralized systems [41], [42], leads to a tendency of urban water managers to first consider extraction from urban rivers and drains when identifying urban stormwater harvesting projects (e.g. [43], [44]). Such a conception of stormwater harvesting has led to a misconception that urban stormwater runoff has some environmental flow benefit [45]. Studies showing consistent degradation in the face of increasing urban stormwater drainage [9], [10] indicate that the reverse is true. Urban stormwater runoff, delivered through conventional drainage systems, is a complex environmental flow problem that can, in large part, be solved by harvesting stormwater *before* it reaches aquatic ecosystems.

A recent review of large-scale urban stormwater harvesting projects funded in Australia [46] showed that almost all projects extracted water from waterways or large drains (rather than directly harvesting from impervious areas). Such schemes can at best harvest a small proportion of the damaging stormwater flows (their ability to divert and store flows is small compared to the large flow rates that accumulate with catchment area), do nothing to restore lost dry-weather flows, and fail to protect upstream waters. Of greater concern, about a quarter of the projects identified harvested dry-weather flows from drains or waterways. These projects only serve to exacerbate the reduction of dry-weather flows caused by reduced infiltration, as observed in this study.

The protection and restoration of urban streams has thus been hampered by a lack of understanding of the unique nature of urban stormwater runoff as an environmental flow problem that could be solved by using stormwater as a water resource. Stormwater harvesting defies the dominant conception of water resource management that extraction of water from ecosystems must result in a monotonic decline in the ecological condition of that ecosystem (Figure 5.I) [47].

By not harvesting stormwater to keep an appropriate proportion of it out of receiving waters, we not only forego the benefits to society of this large water resource, but also contribute to the degradation of waterways (Figure 5.II), resulting in a loss of biodiversity and ecological function provided by healthy streams. In addition, the use of stormwater for landscape irrigation can mitigate against the urban heat island effect [48], [49]. The wider use of urban stormwater would reduce demand on potable water supplies, potentially freeing water to provide environmental flows below water supply storages. In growing cities, urban stormwater harvesting could allow water managers to avoid or delay the augmentation of other potable water supply options that have negative environmental impacts. Retention and use of stormwater also contributes to mitigation of urban flooding, thus potentially reducing costs for flood protection [50].

Using a volume of stormwater equivalent to the volume lost to evapotranspiration in pre-urban state (Figure 5.II B), if coupled with infiltration to restore lost sub-surface flows, provides maximum environmental benefit, by optimizing the retention of water in the catchment and restoration of downstream flow regimes. The integration of harvesting with baseflow restoration techniques such as infiltration is thus important. In contrast, using all available stormwater runoff (Figure 5.II C) has an environmental cost by reducing subsurface flow delivery to stream. However, in many urban settings this loss can be compensated by increased infiltration in non-treed open spaces, or by leakage of water supply systems (e.g. [51]).

Degradation of stream biotic assemblages occurs at very low levels of (connected) imperviousness [17], [52]. Therefore, protection of the ecological integrity of stream ecosystems is likely to require interception and treatment of runoff from almost all catchment impervious surfaces, including the prevention of excess runoff from reaching streams. The provision of environmental flow standards for the protection of streams from urban stormwater runoff will therefore require a universal change from conventional stormwater management in any catchment where the decision is made to protect the stream from stormwater impacts (or to remove existing impacts). Such a fundamental change in practice will require the addressing of technical challenges (concerning design standards, available space), economic barriers (costs of construction and maintenance) and social and institutional impediments. Realizing such change will require that managers of rivers, of stormwater drainage systems, and of urban water resources, recognize the rare advantage of urban stormwater as a water resource that can provide both human and environmental benefit.
